# Characterization of SARS‐CoV‐2 Entry Genes in Skeletal Muscle and Impacts of In Vitro Versus In Vivo Infection

**DOI:** 10.1002/jcsm.13705

**Published:** 2025-01-27

**Authors:** Salyan Bhattarai, Eva Kaufmann, Feng Liang, Yumin Zheng, Ekaterina Gusev, Qutayba Hamid, Jun Ding, Maziar Divangahi, Basil J. Petrof

**Affiliations:** ^1^ Meakins‐Christie Laboratories and Translational Research in Respiratory Diseases Program Research Institute of the McGill University Health Centre Montreal Quebec Canada; ^2^ Respiratory Division Department of Medicine McGill University Montreal Quebec Canada; ^3^ Department of Biomedical and Molecular Sciences Queens University Kingston Ontario Canada; ^4^ Sharjah Institute for Medical Research University of Sharjah UAE

**Keywords:** ACE2, atrophy, COVID‐19, diaphragm, viral entry

## Abstract

**Background:**

COVID‐19 has been associated with both respiratory (diaphragm) and non‐respiratory (limb) muscle atrophy. It is unclear if SARS‐CoV‐2 infection of skeletal muscle plays a role in these changes. This study sought to: 1) determine if cells comprising skeletal muscle tissue, particularly myofibres, express the molecular components required for SARS‐CoV‐2 infection; 2) assess the capacity for direct SARS‐CoV‐2 infection and its impact on atrophy pathway genes in myogenic cells; and 3) in an animal model of COVID‐19, examine the relationship between viral infection of skeletal muscle and myofibre atrophy within the diaphragm and limb muscles.

**Methods:**

We used in silico bioinformatics analysis of published human single cell RNA‐seq datasets, as well as direct qPCR examination of human myotubes and diaphragm biopsies, to assess expression of key genes involved in SARS‐CoV‐2 cellular entry. In Vitro, we determined the ability of SARS‐CoV‐2 to directly infect myogenic cells and employed qPCR to assess the impact on muscle atrophy pathway genes (ubiquitin‐proteasome, autophagy). In vivo, the diaphragm and quadriceps of Roborovski hamsters with SARS‐CoV‐2 respiratory infection were examined at day 3 post‐inoculation to evaluate the relationship between atrophy pathway and SARS‐CoV‐2 transcripts by qPCR, as well as histological measurements of myofibre morphology.

**Results:**

Angiotensin converting enzyme 2 (ACE2), the primary receptor for SARS‐CoV‐2, as well as cooperating proteases (furin, cathepsins B and L), are expressed by myofibres. ACE2 expression was increased 5‐fold (*p* = 0.01) in the diaphragms of mechanically ventilated human subjects compared to controls. In Vitro, a time‐dependent increase of SARS‐CoV‐2 transcript levels was observed in myotubes directly exposed to the virus (*p* = 0.002). This was associated with downregulation of the ubiquitin ligase MuRF1 (by 64%, *p* = 0.002) and the autophagy gene LC3B (by 31%, *p* = 0.009). In contrast, in vivo infection led to upregulation of MuRF1 in quadriceps (23‐fold, *p* = 0.0007) and autophagy genes in both quadriceps (5.2‐fold for Gabarapl1, *p* = 0.03; 7‐fold for p62, *p* = 0.0002) and diaphragm (2.2‐fold for Gabarapl1, *p* = 0.03; 2.3‐fold for p62, *p* = 0.057). In infected hamsters the diaphragm lacked viral transcripts but exhibited atrophy (48% decrease in myofibre area; *p* = 0.02), whereas the quadriceps lacked myofibre atrophy despite elevated viral transcripts in the muscle.

**Conclusions:**

Although myogenic cells express the genes required for SARS‐CoV‐2 entry and can be directly infected, there was no evident relationship between viral transcript levels and manifestations of atrophy, either in vitro or in vivo. Our results do not support direct myofibre infection by SARS‐CoV‐2 as a likely cause of atrophy in COVID‐19.

## 1 Introduction

Coronavirus disease 2019 (COVID‐19), resulting from infection by severe acute respiratory syndrome coronavirus 2 (SARS‐CoV‐2), has a wide spectrum of clinical manifestations ranging from mild symptoms to rapidly progressive multi‐organ failure and death. A substantial proportion of patients with COVID‐19 undergo hospitalization and may require respiratory support with mechanical ventilation [1]. Acutely, both biochemical and histological evidence of skeletal muscle pathology have been reported in patients with COVID‐19 [2]. Furthermore, patients often complain of persistent exercise intolerance and skeletal muscle weakness lasting weeks or months after resolution of the acute illness, which encompasses the syndrome of “long COVID” [3, 4, 5]. Objectively measured weakness of the diaphragm has also been reported in patients previously hospitalized for COVID‐19 [6]. In addition to viral infection per se, factors such as prolonged muscle inactivity, hypoxemia, malnutrition, and certain medications (e.g., corticosteroids) could account for skeletal muscle dysfunction in many patients. Overall, the aetiology and cellular processes underlying skeletal muscle dysfunction in COVID‐19, as well as the direct role of the virus itself in producing these changes, remain unclear.

One plausible mechanism of skeletal muscle dysfunction in SARS‐CoV‐2 infection is systemic mediator release from the lungs and other infected organs (“cytokine storm”). For example, sustained elevation of IL‐6 can cause skeletal muscle atrophy [7] and is associated with worse clinical outcomes in COVID‐19 [8]. Another proposed mechanism is that SARS‐CoV‐2 could have adverse effects on muscle function through direct viral infection of mature skeletal muscle cells, also known as myofibres. In this regard, there are reports of SARS‐CoV‐2 RNA or viral particles being detected within the myofibres of patients with COVID‐19 [9, 10]. On the other hand, other investigators found no evidence of direct myofibre infection by SARS‐CoV‐2 despite the presence of muscle tissue pathology [11, 12, 13]. The discrepancies between these studies could be due to a number of factors including variations in the viral exposure level or timing of muscle sample procurement after infection, as well as the use of different methods to establish the presence of muscle tissue infection.

The entry receptor for SARS‐CoV‐2, angiotensin converting enzyme 2 (ACE2), is bound by the spike (S) protein of the virus [14]. Efficient SARS‐CoV‐2 infection requires proteolytic cleavage of the S protein to allow membrane fusion and virus entry into host cells, which is primarily achieved by transmembrane protease serine 2 (TMPRSS2) in the lungs [15]. In cell types lacking TMPRSS2, SARS‐CoV‐2 infection can still occur via spike protein cleavage by alternative proteases such as furin and cathepsins [14, 15]. To our knowledge, the susceptibility of differentiated skeletal muscle cells to SARS‐CoV‐2 infection has not been directly examined. In addition, the relationship between SARS‐CoV‐2 infection of skeletal muscle tissue and the development of myofibre atrophy is unknown. In the present study, we employed a combination of in vivo (hamster and human muscle tissue), in vitro (differentiated human skeletal muscle cells), and in silico bioinformatics analyses (publicly available human datasets) to address these questions. The results of our investigation provide new insights into the impact of SARS‐CoV‐2 infection on both respiratory (diaphragm) and non‐respiratory skeletal muscles.

## 2 Materials and Methods

### 2.1 In Silico Bioinformatics Analysis

For the non‐myofibre cell populations within skeletal muscle tissue, the cell type‐specific expression of genes associated with SARS‐CoV‐2 infection were evaluated through bioinformatics analyses of two previously published single cell RNA‐seq datasets [16, 17]. We downloaded the pre‐processed single cell datasets of human skeletal muscle tissue mononuclear cells and their cell type annotations from GSE143704 and the Human Cell Atlas Data Explorer (https://explore.data.humancellatlas.org/projects/a39728aa‐70a0‐4201‐b0a2‐81b7badf3e71), respectively. One ten‐patient dataset encompasses 22 058 cells and 21 703 genes, while the other four‐sample dataset contains 2876 cells and 15 408 genes. We employed Scanpy (v1.9.3) (S1) for the standard single cell data analysis and retained all cells and genes, as the datasets were already cleaned in the previous studies. We combined closely related cell type annotations from the original studies (see Figure S1) to enhance clarity and facilitate inter‐dataset comparison. Gene expression levels of ACE2, TMPRSS2, furin, cathepsin B, and cathepsin L across different cell types in each dataset were visualized and examined using dot plots.

For the mature myofibre populations within skeletal muscle tissue, we used the feature count matrix from GSE130977, a bulk RNA‐seq dataset comprising 18 samples comprised of both type I and type IIa myofibres [17], to perform a fibre type‐specific analysis of the same genes. We normalized these data by adjusting for library size variations and performing a logarithmic transformation with an added pseudo‐count of one. Following this initial normalization step, a z‐score normalization was applied across all samples to facilitate comparative analysis using SciPy (v1.10.0) (S2). A heatmap was generated to visualize the expression levels of ACE2, TMPRSS2, furin, cathepsin B, and cathepsin L, revealing expression profiles across the individual samples and fibre types. The scripts and datasets for replicating these results are accessible at our GitHub repository: https://github.com/mcgilldinglab/single_cell_human_muscle_analysis.

### 2.2 Human Lung and Diaphragm Samples

Human tissue sample procurement protocols were approved by the Human Research Ethics Board of the McGill University Health Centre in accordance with the standards of the 1964 Declaration of Helsinki and its later amendments. The clinical and demographic characteristics of human subjects from whom lung or diaphragm tissue samples were obtained are shown in Table S1. Human primary bronchial epithelial cells were obtained from the Biobank of the Quebec Respiratory Health Research Network (https://rsr‐qc.ca/) as we have previously described (S3). Control diaphragm (*n* = 8) and lung (*n* = 6) specimens were obtained in the operating room during elective thoracic surgery for benign or malignant lung lesions. For mechanically ventilated patients, diaphragm biopsies were obtained from critically ill patients diagnosed with brain death who had undergone mechanical ventilation in the intensive care unit for variable periods of time (range of 29 to 93 h; *n* = 7). The diaphragm specimens from these patients were removed in the operating room at the time of organ donation harvest as we have previously described (S4, S5). All specimens were frozen in liquid nitrogen and stored at −80 °C prior to the COVID‐19 pandemic.

### 2.3 Generation and Quantification of SARS‐CoV‐2 Virus

All experiments involving infectious SARS‐CoV‐2, both in vivo and in vitro, were performed in the Biosafety Level (BSL)‐3 facility of the Research Institute of the McGill University Health Centre (Montreal, Quebec, Canada). SARS‐CoV‐2/RIM‐1 (formerly known as SARS‐CoV‐2/cp13.32, lineage B.1.147) was used for all experiments (S6). Propagation of SARS‐CoV‐2 and collection of virus‐containing supernatant were performed in cultures of confluent VeroE6 cells, a kidney epithelial cell line permissive to SARS‐CoV‐2 infection. The virus was titrated using a standard median tissue culture infectious dose (TCID)‐ 50 assay. The presence of SARS‐CoV‐2 within cultured cells or tissues was assessed by PCR quantification of the Upstream from the Envelope (UpE) transcript between the ORF3a and E gene regions of the viral genome as previously described [18] (S3).

### 2.4 Infection of Skeletal Muscle Cells in Vitro

Immortalized human skeletal muscle cells were kindly provided by Dr. Eric Shoubridge (Montreal Neurological Institute, McGill University) (S7). Myoblasts were allowed to proliferate in culture media (SK Max kit, Wisent Bioproducts, Quebec, Canada) containing 15% foetal bovine serum without antibiotics. After reaching 90% confluency the myoblasts were switched to 2% horse serum for 5 days to induce differentiation into multinucleated myotubes. Myotubes were infected with SARS‐CoV‐2 at multiplicity of infection (MOI) = 0.7 on day 5 of differentiation. The MOI calculation was based on the numbers of myoblasts present in the wells before switching to differentiation medium. Two experimental conditions were studied: 1) 4 h of infection followed by incubation with virus‐free media for another 20 h prior to cell harvesting; 2) 24 h of infection followed by cell harvesting. Myotubes incubated with virus‐free medium served as a negative control; primary human bronchial epithelial cells cultured as we have previously described (S3) were infected at MOI = 0.35 for 4 h and used as a positive control.

### 2.5 Infection of Hamsters in Vivo

All animal protocols were approved by the McGill University Health Centre Animal Care Committee, and conducted in accordance with regulations set forth by the Canadian Council on Animal Care and the ARRIVE guidelines. Roborovski dwarf hamsters (*Phodopus roborovski*; *n* = 8), which have natural susceptibility to severe SARS‐CoV‐2 infection [19, 20], were obtained from a pet shop trade breeder at 3–4 weeks of age. The animals were clinically examined, tested for pathogens, and then housed under specific pathogen‐free conditions. Roborovski hamsters were intranasally instilled with either PBS or a sublethal dose of SARS‐CoV‐2 (1.4 × 10^4^ plaque forming units) as we have previously described [18]. The experiments were performed at 8–12 weeks of age with the two groups being age‐ and sex‐matched. The hamsters were sacrificed on day 3 post‐infection based on previous findings in this model showing that the animals lose 10%–15% of their body weight and the pulmonary viral load is maximal [18, 20].

### 2.6 Quantitative Reverse Transcription PCR (RT‐PCR)

Harvested cells or tissues were immediately placed in TRIzol reagent (Fisher Scientific, USA) and then stored at −80°C. Total RNA was later extracted according to the manufacturer's instructions. Following DNase I treatment (Gibco, USA), 1 μg of RNA was reverse transcribed (Superscript II, Invitrogen, USA) using random primers for cDNA generation. Quantitative PCR (qPCR) was performed using 10 ng of cDNA mixed with 5 μL of 2× SYBR Green (Invitrogen, USA) and 0.5 μL of 10 μM primer mixes for 40 cycles (StepOne Plus Thermocycler, Applied Biosystems, USA). The β‐actin gene was used as an internal control housekeeping gene and was confirmed to be stable across the different experimental conditions. Relative mRNA level quantification was determined using the 2^‐△△Ct^ method, expressed as n‐fold change from the mean control group value. All PCR primer sequences are provided in Table S2.

### 2.7 Histological Analysis

Excised tissues were immediately fixed and maintained in 10% formalin at room temperature for 48 h before paraffin embedding and sectioning (10 μm thickness). Haematoxylin and eosin as well as agglutinin staining were performed. For the latter the muscle sections were deparaffinized, permeabilized with 0.1% Triton‐X 100, and then stained with Wheat Germ Agglutinin Alexa Fluor 488 Conjugate (1:1000 dilution; Invitrogen, USA) overnight at 4°C. Images were acquired with a Zeiss Axio Imager M2 microscope. The following quantitative measurements of individual myofibre size and morphology were determined: 1) myofibre cross‐sectional area, 2) myofibre roundness (roundness index = 4Pi × fibre area/fibre perimeter^2^), and 3) myofibre area variability (variance coefficient = 1000 × standard deviation of fibre area/mean fibre area). The mean number (±SE) of fibres analysed was 277 (±51) and 489 (±93) for the diaphragm and quadriceps muscles, respectively. The measurements were performed by a single operator blinded to sample identity using ImageJ software.

### 2.8 Statistics

#### 2.8.1 In Silico Bioinformatics Analyses

For the single cell RNA‐seq datasets which have a heavily skewed distribution (S8), comparisons of gene expression in the non‐myofibre cell populations employed SciPy (S2) to perform a non‐parametric permutation test with 10 000 random permutations (S9). For the bulk RNA‐seq dataset used for comparisons of gene expression in the type I and type IIa myofibre populations, Student's *t*‐test was applied. For all of the above bioinformatics analyses, the False Discovery Rate (FDR) with Benjamini–Hochberg procedure was used to correct for multiple comparisons, and gene pairs with an adjusted FDR < 0.05 were considered to be significantly different.

#### 2.8.2 In Vitro and In Vivo Analyses

For all cell culture, in vivo tissue and animal data, statistical analysis was performed using Prism GraphPad (v8.0). Distribution of the data was first assessed using the Shapiro–Wilk normality test. For normally distributed data, comparisons between two groups were made using Student's unpaired *t*‐test; comparisons between more than two groups were made with ANOVA followed by post‐hoc application of the Tukey test to adjust for multiple comparisons. For non‐normally distributed data, comparisons between two groups were made using the Mann–Whitney U test, while comparisons between more than two groups were made with the Kruskal–Wallis test followed by post‐hoc application of Dunn's test. All graphs show group mean data and standard error (SE) unless specified otherwise. Statistical significance was defined as *p* < 0.05 (two‐tailed) in all cases.

## 3 Results

### 3.1 Genes Required for SARS‐CoV‐2 Infection Are Expressed in Skeletal Muscle Cells Both In Vivo and In Vitro

We first evaluated expression levels of the genes required for SARS‐CoV‐2 entry in the different cell types which constitute human skeletal muscle tissue, by performing a bioinformatics analysis of previously published RNA‐seq datasets. The analysis included different non‐myofibre mononuclear cell populations (fibroblasts, endothelial cells, etc.) found within skeletal muscle tissue as well as muscle stem (satellite) cells and isolated mature single myofibres. In addition to the ACE2 receptor, we assessed the following proteases, which are capable of SARS‐CoV‐2 spike protein cleavage: TMPRSS2, furin, cathepsin B, and cathepsin L (see Supplemental Data File for individual FDR values).

**FIGURE 1:**
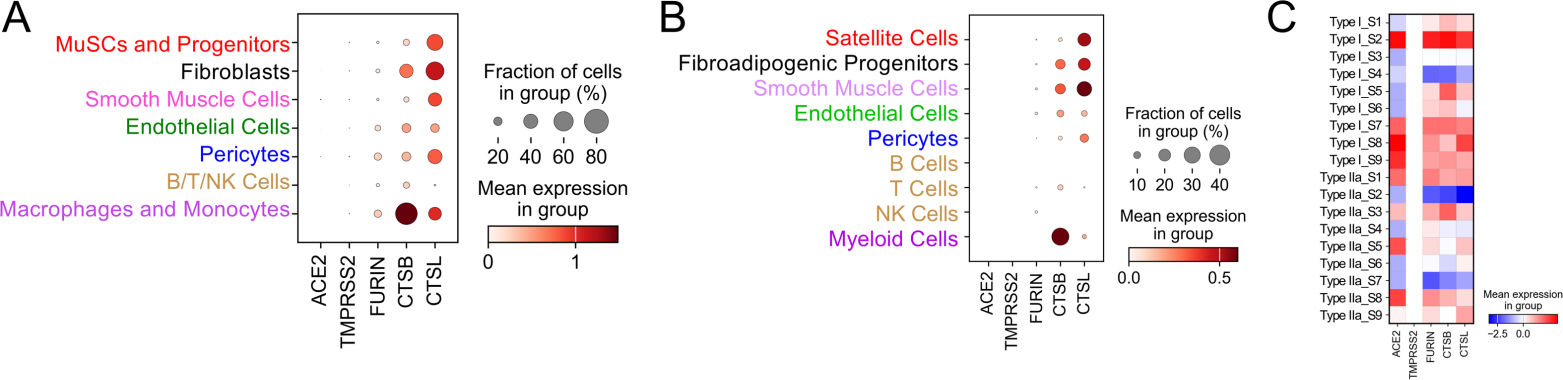
Bioinformatics analysis of SARS‐CoV‐2 entry gene expression in human skeletal muscle. (A, B) Dot plots depict the expression of SARS‐CoV‐2 entry genes in different mononuclear cell types in two separate human skeletal muscle single cell RNA‐seq datasets (panel A is from reference [16] and panel B is from reference [17]) using combined annotations from the original studies (see Figure S1 for original source dataset annotations). Circle size represents the proportion of cells expressing each gene, while colour intensity indicates gene expression levels. The related cell types in the two datasets are uniformly coloured to facilitate comparisons. (C) Heatmap of z‐score normalized gene expression levels based on bulk RNA‐seq data obtained from isolated single (S) myofibre samples of either slow‐twitch type I or fast‐twitch type IIa phenotype (from reference [17]). Abbreviations: CTSB, cathepsin B; CTSL, cathepsin L; MuSCs, muscle stem cells; TMPRSS2, transmembrane protease serine 2.

For the non‐myofibre mononuclear cell populations within human skeletal muscle tissue (Figure 1A,B), the two separate single cell RNA‐seq datasets both showed that ACE2 and furin transcripts were either undetectable or at a low level in all cell types, whereas cathepsins B and L were more highly expressed. TMPRSS2 transcripts were very low or undetectable in all of the non‐myofibre cell types examined. For the isolated single myofibres (Figure 1C), ACE2 as well as furin, cathepsin B, and cathepsin L were substantially expressed while TMPRSS2 was not detected. This pattern was present in both type I (slow‐twitch) and type IIa (fast‐twitch) fibres, and there was no significant differential gene expression between the two fibre types. Taken together, the above findings suggest that in skeletal muscle tissue, myofibres themselves are a principal site of expression for the SARS‐CoV‐2 entry receptor ACE2, as well as spike protein‐activating proteases with the exception of TMPRSS2.

**FIGURE 2:**
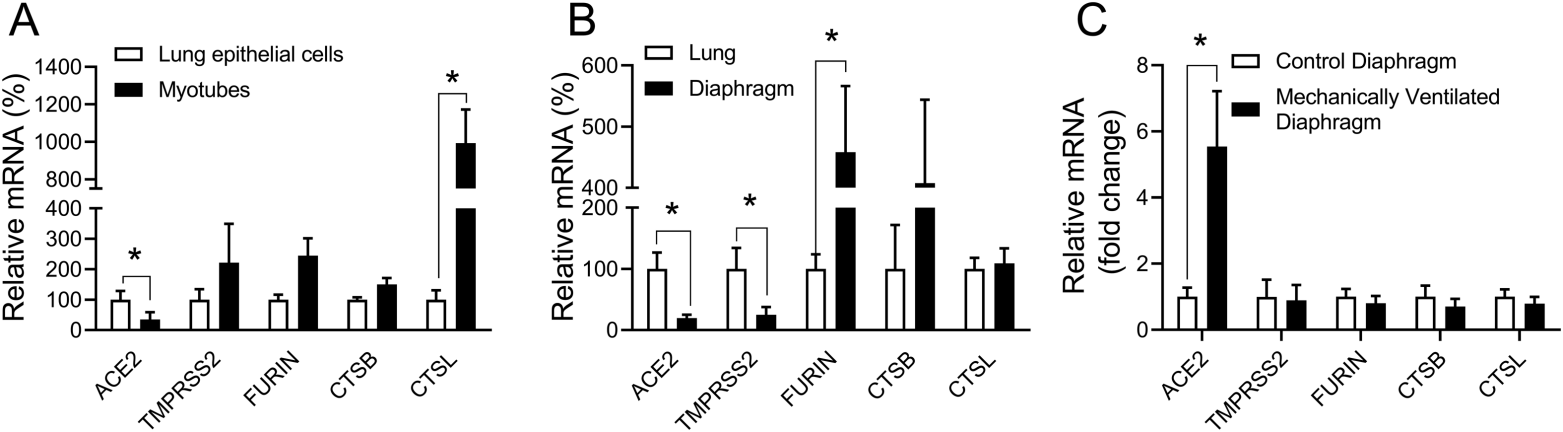
Expression of SARS‐CoV‐2 entry genes in human myotubes and diaphragm biopsies. Transcript levels of genes required for cellular entry of SARS‐CoV‐2 were determined by qPCR in: (A) myotubes and lung epithelial cells in vitro (myotubes *n* = 8, lung epithelial cells *n* = 5), (B) human diaphragm and lung biopsies (diaphragm: *n* = 8; lung: *n* = 6) and (C) diaphragm biopsies from mechanically ventilated intensive care unit (ICU) patients (*n* = 7) and elective thoracic surgery controls (*n* = 8). For (A) and (B), values are expressed as a percentage of the mean lung specimen value. For (C), values are expressed as n‐fold change relative to the mean control group value. Group means and SE bars are illustrated. *p < 0.05 between indicated groups.

To build on these observations, we next directly compared expression levels of the above genes between human skeletal muscle and lung cells, since the latter are the primary target of SARS‐CoV‐2 infection. In cultured cells, ACE2 expression was lower in human myotubes than in human lung epithelial cells (*p* = 0.048), cathepsin L transcript levels were higher in myotubes (*p* = 0.003), and the other genes did not demonstrate significant differences (Figure 2A). In whole tissue biopsy samples obtained from human control subjects, the mean values for ACE2 and TMPRSS2 expression in the diaphragm were on average only 19% (*p* = 0.005) and 25% (*p* = 0.04) of lung tissue levels, respectively (Figure 2B). The other genes were expressed in human diaphragm tissue at levels that were either greater (furin, *p* = 0.02) or not significantly different (cathepsins B and L) from the values found in human lung biopsy specimens.

Because patients with severe SARS‐CoV‐2 pulmonary infection can require the use of mechanical ventilation, we also evaluated the expression of viral entry genes in diaphragm biopsies obtained from mechanically ventilated patients with critical illness of various aetiologies (see Table S1). Transcript levels of human ACE2 were substantially increased (approximately 5‐fold higher) in diaphragms of mechanically ventilated patients with critical illness compared to controls (*p* = 0.01), whereas expression of the other SARS‐CoV‐2 entry genes did not differ significantly from control levels (Figure 2C).

### 3.2 In Vitro Effects of SARS‐CoV‐2 on Human Myotubes

**FIGURE 3:**
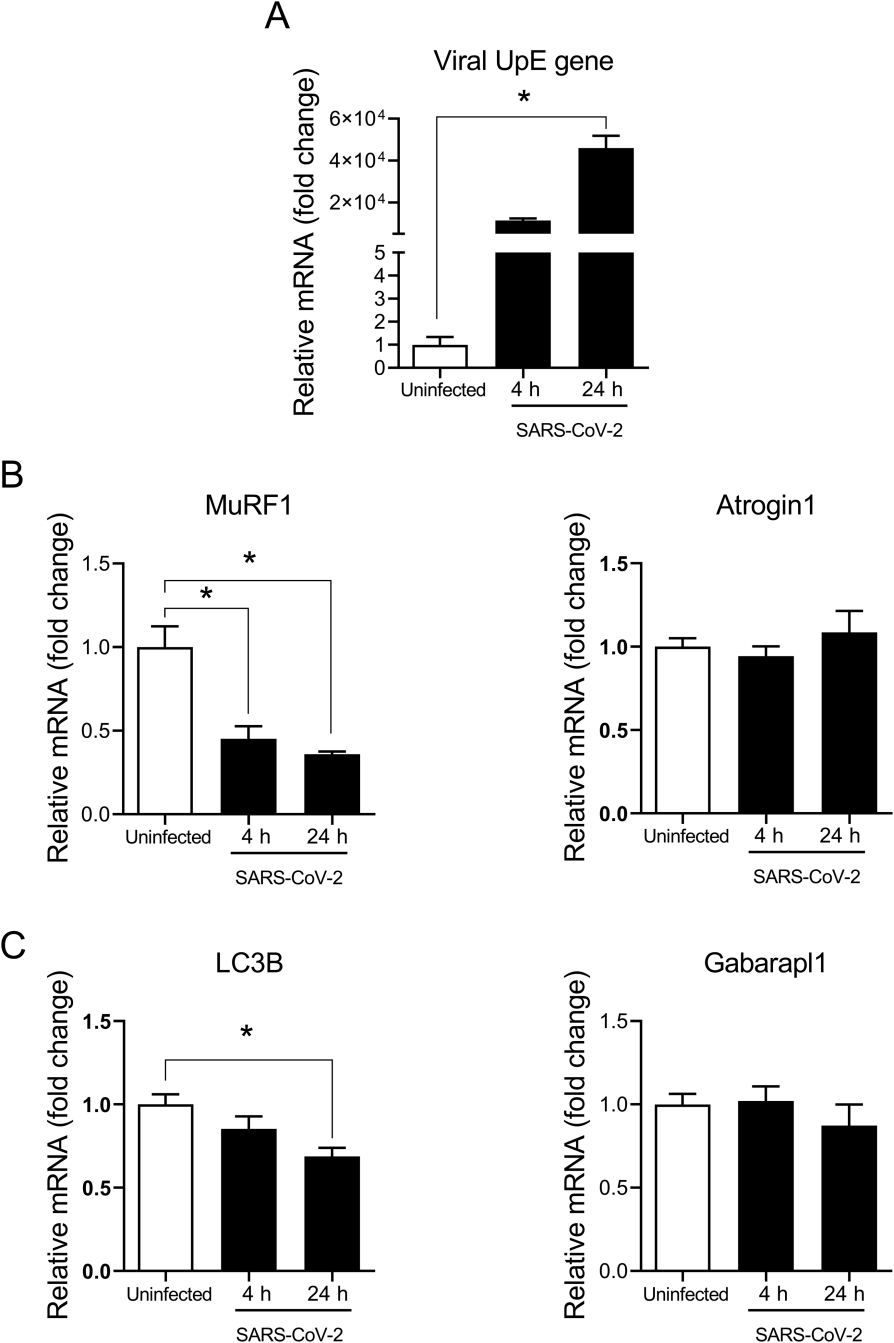
Impact of SARS‐CoV‐2 on expression of viral transcripts and muscle atrophy genes in cultured myotubes. At 4 h and 24 h after exposure of differentiated human myotubes to SARS‐CoV‐2, transcript levels were determined by qPCR for the following: (A) viral UpE, (B) E3 ubiquitin ligases and (C) autophagy‐related genes. All mRNA data are expressed as n‐fold change relative to the mean value in uninfected myotubes. Group means and SE bars are illustrated (n = 6 per group). *p < 0.05 between indicated groups.

To determine whether SARS‐CoV‐2 is able to directly infect human skeletal muscle cells, the UpE viral gene transcripts of SARS‐CoV‐2 were measured by qPCR in differentiated myotubes directly exposed to the virus in vitro. In keeping with progressive infection, viral gene transcript levels rose in a time‐dependent manner in myotubes incubated with SARS‐CoV‐2 (*p* = 0.002 at 24 h) (Figure 3A). Using the same in vitro system, we also measured transcript levels of E3 ubiquitin ligases (MuRF1, Atrogin1) and autophagy‐related genes (LC3B, Gabarapl1) previously implicated in muscle atrophy. In the human myotubes exposed to SARS‐CoV‐2, decreased expression levels of the E3 ubiquitin ligase MuRF1 (*p* = 0.002 at 24 h) (Figure 3B) as well as the autophagy‐related gene LC3B (*p* = 0.009 at 24 h) (Figure 3C) were observed in comparison to uninfected cells.

### 3.3 In Vivo Effects of Pulmonary Infection With SARS‐CoV‐2 on Diaphragm and Limb Muscles

**FIGURE 4:**
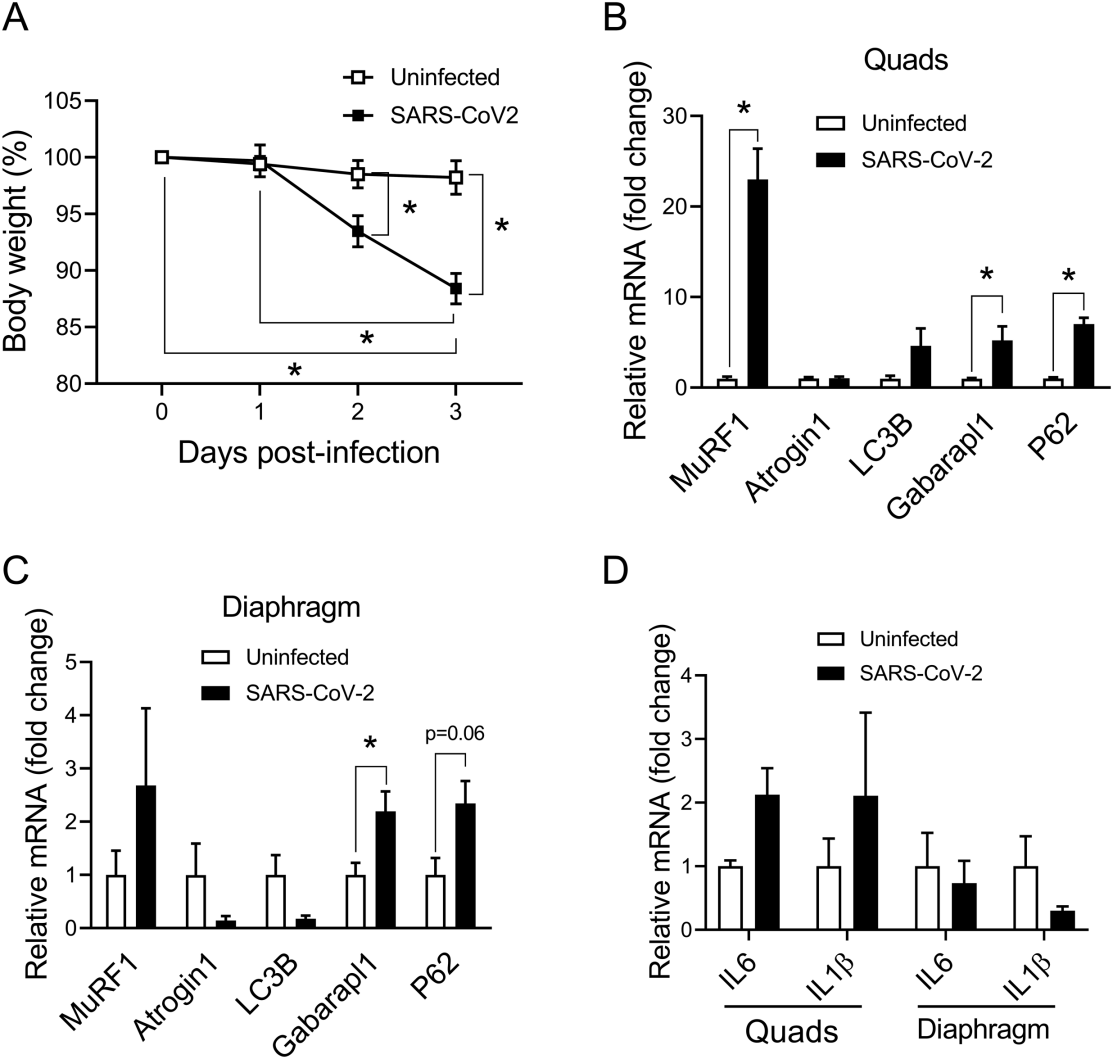
Effects of pulmonary infection on body weight and muscle atrophy gene expression in Roborovski hamsters. (A) Hamster body weights at different time points post‐infection are shown as a percentage of the initial pre‐infection value. In (B) quadriceps and (C) diaphragm, expression levels of prototypical muscle atrophy genes from the ubiquitin‐proteasome (MuRF1, Atrogin1) and autophagy (LC3B, Gabarapl1, p62) systems were determined at 3 days post‐infection. (D) Expression levels of pro‐inflammatory cytokines (IL‐6, IL‐1b) were determined at the same time point in both muscles. All transcript data are expressed as n‐fold change relative to the mean value in uninfected animals. Group means and SE bars are illustrated (*n* = 4 per group). *p < 0.05 between indicated groups.

To assess the in vivo impact of SARS‐CoV‐2 pulmonary infection on skeletal muscles, hamsters which had received a sublethal inoculation of SARS‐CoV‐2 were compared to uninfected hamsters on day 3 post‐infection. Initial baseline body weights did not differ significantly between the infected and uninfected animals (16.50 ± 0.69 g and 17.44 ± 1.24 g, respectively). However, on day 3 body weight had decreased by approximately 11% in the infected group (*p* = 0.001), whereas no significant change occurred in uninfected hamsters (Figure 4A). Expression levels of the E3 ubiquitin ligase MuRF1 and/or the autophagy‐related genes Gabarapl1 and p62 were greater in the quadriceps (Figure 4B) and diaphragm (Figure 4C) muscles of infected animals on day 3 post‐infection. However, there were no significant changes in expression levels of the pro‐inflammatory cytokines, IL‐6 and IL‐1β, in either muscle following infection (Figure 4D).

**FIGURE 5:**
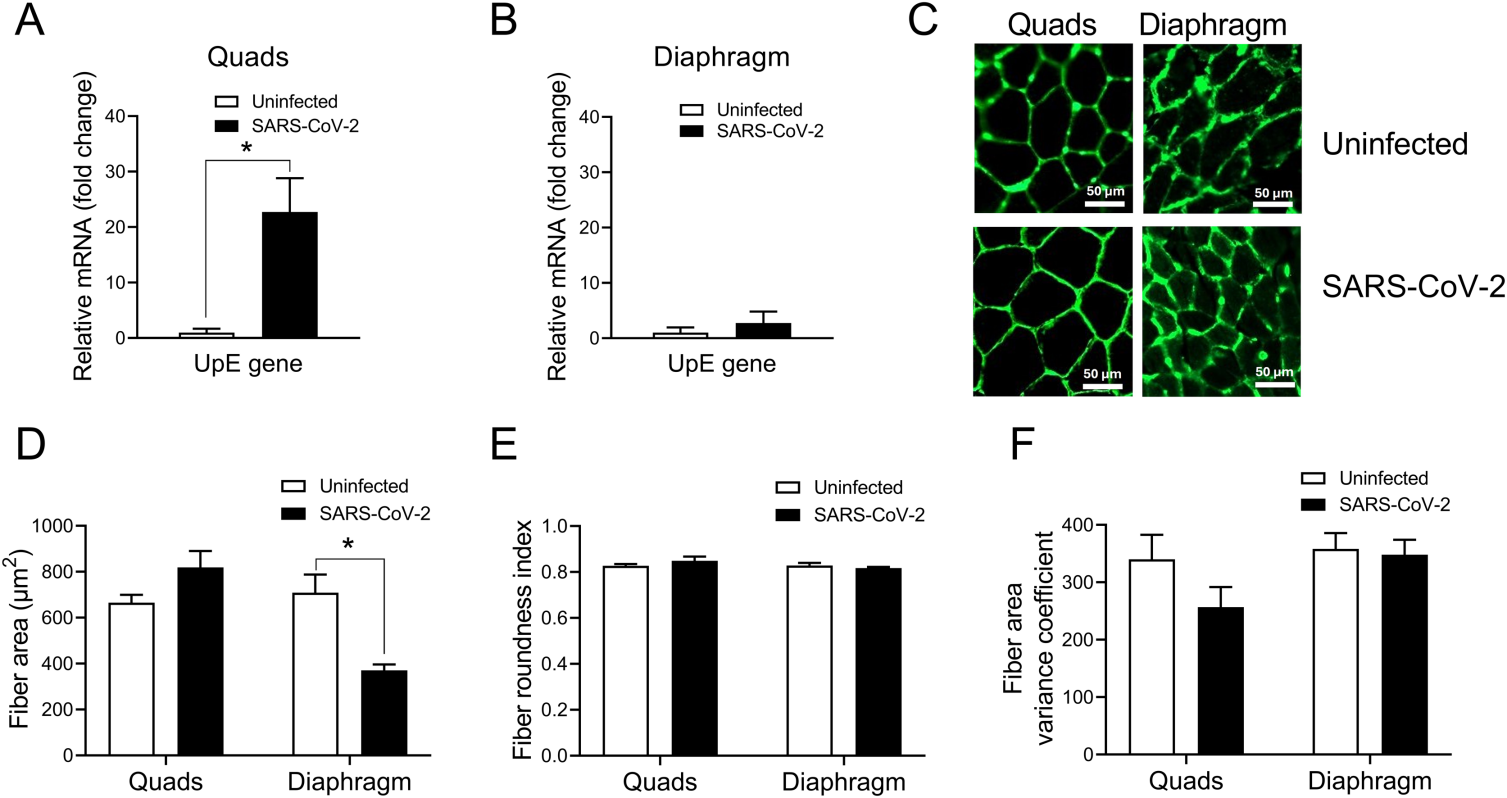
SARS‐CoV‐2 transcript levels and myofibre morphology in quadriceps and diaphragm following pulmonary infection. In (A) quadriceps and (B) diaphragm, UpE viral transcript levels are shown at 3 days post‐infection. (C) Representative images of agglutinin‐stained muscles at the same time point are shown. Muscle morphology measurements consisted of: (D) mean individual myofibre cross‐sectional area, (E) degree of myofibre roundness, and (F) variability (variance coefficient) of myofibre area. Viral transcript data are expressed as n‐fold change relative to the mean background signal obtained in uninfected animals. Group means and SE bars are illustrated (*n* = 4 per group). *p < 0.05 between indicated groups.

Viral gene transcript levels were determined by qPCR in the quadriceps and diaphragm at the same time point after pulmonary infection. UpE transcript levels were increased in the quadriceps muscle on day 3 post‐infection (*p* = 0.01) (Figure 5A). Despite this increase in viral transcript levels, mean myofibre size (cross‐sectional area) was not significantly altered in the quadriceps (Figure 5C,D). In diaphragms of the same infected hamsters, there was no significant increase of UpE transcripts above the non‐specific background signal found in uninfected control animals (Figure 5B). Nevertheless, the diaphragm exhibited lower values for myofibre cross‐sectional area after infection in comparison to the uninfected controls (*p* = 0.02) (Figure 5C,D). There were no other differences in myofibre morphology between infected and uninfected animals in either muscle, as assessed by the degree of myofibre roundness (Figure 5E) and the variability of myofibre size (Figure 5F).

## 4 Discussion

In the present study, we performed an in‐depth analysis of SARS‐CoV‐2 entry genes and the acute biological effects of viral infection in skeletal muscle, both in vitro and in vivo. The main findings of our investigation can be briefly summarized as follows. First, in silico bioinformatics analysis of published human RNA‐seq data as well as our own direct measurements of transcript levels indicated that ACE2, the primary receptor for SARS‐CoV‐2 infection, as well as its cooperating proteases (i.e., furin and cathepsins), are substantially expressed by myofibres. Second, viral transcripts were increased in differentiated skeletal muscle cells (myotubes) directly exposed to the virus in vitro, as well as in the quadriceps muscle tissue of animals infected in vivo through the respiratory route. Third, although muscle atrophy pathway genes (ubiquitin‐proteasome, autophagy) were downregulated in myotubes directly exposed to SARS‐CoV‐2 in vitro, these genes were upregulated to various degrees in the muscle tissues of animals infected in vivo. Finally, there was no apparent relationship between detection of SARS‐CoV‐2 transcripts in the muscle tissue of infected animals and the presence of myofibre atrophy.

Before discussing these results in detail, certain limitations of our study should be acknowledged. Firstly, we employed the Roborovski hamster strain due to its high susceptibility to SARS‐CoV‐2 infection [19, 20]. Based on prior work indicating that humane endpoints (weight loss, general status) and high viral loads in multiple organs (lungs, jejunum, kidneys) are often attained at this time point [20] as well as the limited availability of these animals, we focused our in vivo experiments on day 3 post‐infection. Although myofibre atrophy was not observed in the quadriceps muscle at this time point, we cannot exclude the possibility that some loss of myofibrillar protein occurred as a precursor to histological atrophy. Secondly, we were unable to directly assess muscle contractility due to the local institutional requirement to perform all of our experiments within the Biosafety Level 3 facility, hence our outcomes are limited to biochemical or histological analyses. Thirdly, our study employed a human muscle cell line and we did not assess the susceptibility to infection of non‐myofibre cell types in vitro or whether results might vary using other strains of SARS‐CoV‐2 [21].

It has been unclear from previous studies if direct infection of myofibres with SARS‐CoV‐2 can occur and whether this plays a significant role in the skeletal muscle abnormalities reported in patients with COVID‐19. For example, in small case series of human subjects, detection of viral RNA by PCR in whole skeletal muscle tissues has been reported in 5%–16% of patients [10, 12]. Some investigators have also observed signs of SARS‐CoV‐2 within individual myofibres [9, 10]. In contrast, other studies found no evidence of skeletal muscle SARS‐CoV‐2 infection despite signs of muscle fibre pathology [11, 13]. A fundamental and previously unanswered question is the extent to which myofibres and other cell types contained within skeletal muscle tissue express the molecular components required for cellular entry of SARS‐CoV‐2. In addition to ACE2 which acts as the primary receptor, SARS‐CoV‐2 entry into cells also requires host proteases to cleave and activate its spike (S) protein which permits fusion of the viral and host cell membranes. Although S protein cleavage is primarily achieved by TMPRSS2 in lung epithelial cells, we found that TMPRSS2 expression is essentially absent in mature myofibres. However, alternative proteases such as furin and lysosomal cathepsins can also perform this function [14, 15, 22]. For instance, due to the presence of furin, cardiomyocytes can be directly infected by SARS‐CoV‐2 despite a lack of TMPRSS2 [23].

We found that ACE2, furin, cathepsin B, and cathepsin L expression are all readily detectable in human skeletal muscle tissue. Furthermore, our bioinformatics analysis suggests that ACE2 and furin are primarily expressed by the myofibres themselves, while cathepsins are more highly expressed by other cell types (e.g., cathepsin B by myeloid cells; cathepsin L by satellite/stem cells and fibroblasts) within whole skeletal muscle tissue. In keeping with our findings in whole tissue biopsies of skeletal muscle, we observed that the genes required for SARS‐CoV‐2 entry are expressed in cultured human myotubes. In addition, we documented a time‐dependent increase of viral transcripts in myotubes exposed to the virus in vitro, showing for the first time the capacity of differentiated skeletal muscle cells to be directly infected by SARS‐CoV‐2.

An intriguing finding of our study is that ACE2 expression was increased in the diaphragms of critically ill (non‐COVID) patients who had undergone mechanical ventilation in the ICU. Although the ACE2 receptor was expressed at a five‐fold lower level in whole diaphragm tissue as compared to the lung under basal conditions, ACE2 was upregulated to approximately the same level as the lung in the diaphragms of these critically ill patients. Whether this was due to the use of mechanical ventilation or other factors associated with critical illness cannot be ascertained. However, these findings are reminiscent of other pathological conditions such as heart failure [24] or inhalational insults to the lung [25, 26], where ACE2 upregulation has also been described. Studies of hospitalized patients with COVID‐19 have reported associations with diaphragm thinning [27], persistent diaphragm contractile dysfunction [6], and fibrotic histopathology in the diaphragm [10]. The relevance of elevated ACE2 expression in the diaphragm as a potential susceptibility factor to these complications remains unknown and will require additional investigation.

To our knowledge, there has been no previous examination of SARS‐CoV‐2 effects on skeletal muscle cells in vitro. Although we originally hypothesized that viral infection would cause an upregulation of prototypical ubiquitin ligases and autophagy system genes associated with muscle atrophy [28], myotubes directly incubated with the virus actually exhibited the opposite response. A similar phenomenon was recently described following SARS‐CoV‐2 infection of cardiomyocytes in vitro, where depression of the ubiquitin‐proteasome system occurred [29]. Infection by SARS‐CoV‐2 also represses ubiquitination of key proteins involved in host defence in other cell types [30, 31] and downregulates autophagy pathway genes in the lung [32]. Accordingly, the downregulation of ubiquitin‐proteasome and autophagy genes in infected skeletal muscle cells is likely part of a generalized strategy employed by SARS‐CoV‐2 to evade the host immune system and promote viral survival [33].

During the acute phase of the illness, mild elevations of blood creatine kinase consistent with skeletal muscle injury are not uncommon and rare cases of severe rhabdomyolysis have also been reported in patients with COVID‐19 [13, 34, 35]. In autopsy studies, an increased prevalence of leukocyte infiltration (e.g., CD8+ T cells and macrophages), myofibre atrophy with preferential loss of thick filaments, mitochondrial abnormalities, autophagic vacuoles, and occasional necrotic or regenerating fibres have been observed in the limb muscles [9, 12, 13]. It is important to recognize that many of the above pathological phenomena described in skeletal muscles of patients with COVID‐19 can occur non‐specifically as manifestations of critical illness myopathy or neuropathy [36]. In the present study, we did not observe histological evidence of muscle necrosis or regeneration in the muscles of infected hamsters. In addition, pro‐inflammatory cytokine gene expression levels were not elevated in the muscles of infected animals.

A notable finding of our study was the lack of any demonstrable relationship between myofibre atrophy and the levels of viral transcripts in the muscles of infected hamsters in vivo. The opposite responses of ubiquitin ligase and autophagy genes to SARS‐CoV‐2 infection in vivo (upregulated) versus in vitro (downregulated) also suggest that myofibre atrophy in vivo was not triggered by direct viral infection of myofibres. We speculate that the different responses of quadriceps and diaphragm with respect to myofibre atrophy are due to a greater vulnerability of the diaphragm to systemic inflammation. Along these lines, Divangahi et al. [37] found that acute bacterial infection of the lungs in mice caused diaphragm weakness without affecting limb muscle function. In rats with polymicrobial sepsis, Lin et al. [38] observed that the diaphragm exhibited greater myofibre injury than the limb muscles. Demoule et al. [39] similarly reported that acute endotoxemia in mice induced higher levels of pro‐inflammatory mediators in the diaphragm than in hindlimb muscle. In a non‐infectious transgenic mouse model with elevated systemic levels of TNF‐α, Li et al. [40] also noted significant diaphragm weakness whereas hindlimb muscle force was preserved. Taken together these studies support the idea that the diaphragm may have a greater inherent susceptibility to the adverse effects of systemic inflammation, which could also be the case during SARS‐CoV‐2 infection.

In summary, we have shown that myofibres express all of the necessary molecular components for SARS‐CoV‐2 entry and this is in keeping with our demonstration that differentiated myotubes can be directly infected by the virus. However, in the common clinical setting of SARS‐CoV‐2 infection introduced by the respiratory route, our data do not support a direct link between myofibre infection by the virus and the development of myofibre atrophy. Instead, our results favour a scenario in which systemic inflammation or other factors associated with SARS‐CoV‐2 pulmonary infection act as the primary driver of myofibre atrophy in COVID‐19. Our findings also suggest that the diaphragm is particularly vulnerable to such systemic factors, which could in turn promote respiratory failure when combined with the increased work of breathing imposed by the virus‐induced pulmonary disease.

## Conflicts of Interest

The authors declare no conflicts of interest.

## Supporting information


**Data S1** Supporting Information.

**Figure S1** Original source study annotations used for bioinformatics analyses.

**Data S2** Supporting Information.

**Table S1** Demographic data and relevant medical history in all human subjects

**Table S2** PCR Primer Sequences
