# Supplementary material for: Characterization of SARS‐CoV‐2 Entry Genes in Skeletal Muscle and Impacts of In Vitro Versus In Vivo Infection
Source: J Cachexia Sarcopenia Muscle. 2025 Jan 27;16(1):e13705. doi: 10.1002/jcsm.13705 (PMC11772215; doi:10.1002/jcsm.13705)
Supplement: Supplementary file 2 — Figure S1 Original source study annotations used for bioinformatics analyses. [file JCSM-16-e13705-s004.docx]

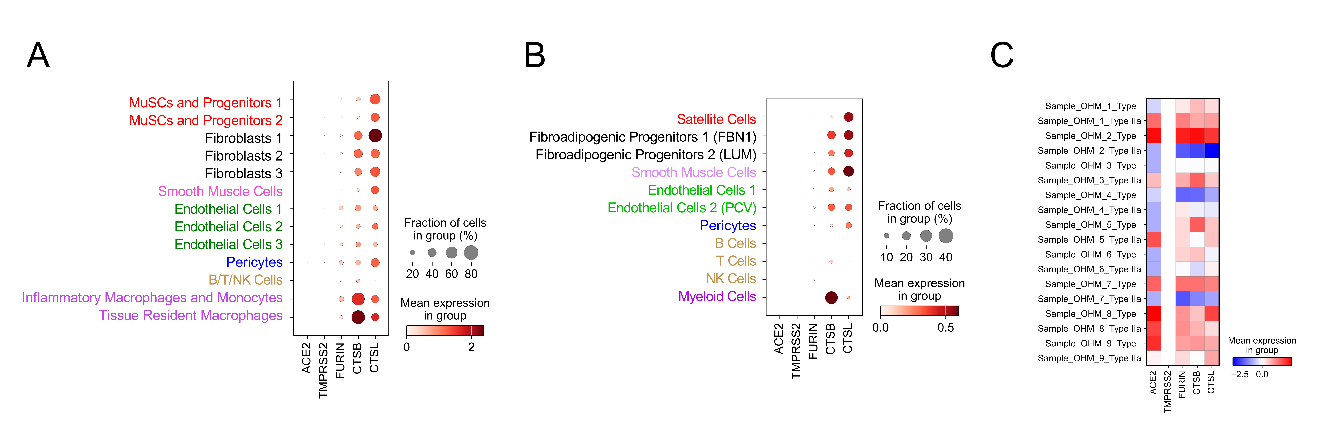


**Supplemental Figure 1: Original source study annotations used for bioinformatics analyses**. (A, B) Dot plots of gene expression in mononuclear cell types from human skeletal muscle based on single cell RNA-seq datasets (panel A is from reference (16) and panel B is from reference (17) ), showing the original cell annotations used in these publications. For Figure 1A we combined the above panel A subgroups of MuSCs and Progenitors, Fibroblasts, Endothelial Cells, and Macrophages; for Figure 1B we combined the above panel B subgroups of Fibroadipogenic Progenitors and Endothelial Cells. (C) Heatmap of z-score normalized gene expression levels in myofiber samples from reference (17), showing the original sample nomenclature used in this publication.
