# Supplementary material for: Characterization of SARS‐CoV‐2 Entry Genes in Skeletal Muscle and Impacts of In Vitro Versus In Vivo Infection
Source: J Cachexia Sarcopenia Muscle. 2025 Jan 27;16(1):e13705. doi: 10.1002/jcsm.13705 (PMC11772215; doi:10.1002/jcsm.13705)
Supplement: Supplementary file 3 — Data S2 Supporting Information. [file JCSM-16-e13705-s002.docx]

**Supplemental References**

S1. Wolf FA, Angerer P, Theis FJ. SCANPY: Large-scale single-cell gene expression data analysis. Genome Biol. 2018 Feb 6;19(1).

S2. Virtanen P, Gommers R, Oliphant TE, Haberland M, Reddy T, Cournapeau D, et al. SciPy 1.0: Fundamental algorithms for scientific computing in Python. Nat Methods. 2020 Mar 1;17(3):261–72.

S3. Gaudet M, Kaufmann E, Jalaleddine N, Mogas A, Hachim M, Senok A, et al. Lung epithelial cells from obese patients have impaired control of SARS-CoV-2 infection. Int J Mol Sci. 2023 Apr 1;24(7).

S4. Picard M, Jung B, Liang F, Azuelos I, Hussain S, Goldberg P, et al. Mitochondrial dysfunction and lipid accumulation in the human diaphragm during mechanical ventilation. Am J Respir Crit Care Med. 2012 Dec;186(11):1140–9.

S5. Hussain SNA, Cornachione AS, Guichon C, Al Khunaizi A, De Souza Leite F, Petrof BJ, et al. Prolonged controlled mechanical ventilation in humans triggers myofibrillar contractile dysfunction and myofilament protein loss in the diaphragm. Thorax. 2016 May 1;71(5):436–45.

S6. Murall CL, Fournier E, Galvez JH, N’Guessan A, Reiling SJ, Quirion PO, et al. A small number of early introductions seeded widespread transmission of SARS-CoV-2 in Québec, Canada. Genome Med. 2021 Dec 1;13(1).

S7. Lochmüller H, Johns T, Shoubridge EA. Expression of the E6 and E7 genes of human papillomavirus (HPV16) extends the life span of human myoblasts. Exp Cell Res. 1999 Apr 10;248(1):186–93.

S8. Watson ER, Mora A, Fard AT, Mar JC. How does the structure of data impact cell-cell similarity? Evaluating how structural properties influence the performance of proximity metrics in single cell RNA-seq data. Brief Bioinform. 2022 Nov 1;23(6).

S9. Nichols TE, Holmes AP. Nonparametric permutation tests for functional neuroimaging: a primer with examples. Hum Brain Mapp. 2002;15(1):1–25.
