# Supplementary material for: Characterization of SARS‐CoV‐2 Entry Genes in Skeletal Muscle and Impacts of In Vitro Versus In Vivo Infection
Source: J Cachexia Sarcopenia Muscle. 2025 Jan 27;16(1):e13705. doi: 10.1002/jcsm.13705 (PMC11772215; doi:10.1002/jcsm.13705)
Supplement: Supplementary file 4 — Table S1 Demographic data and relevant medical history in all human subjects [file JCSM-16-e13705-s003.docx]

**Supplemental Table 1: Demographic data and relevant medical history in all human subjects**

| **Subject** | **Age**  **(yr)** | **Sex** | **MV Duration**  **In ICU (h)** | **Intervention / Diagnosis** | **Other Medical History** |
| --- | --- | --- | --- | --- | --- |
| **MV Diaphragms** | | | | **Organ Donation / Brain death** |  |
| 1 | 65 | M | 32.5 | Cerebrovascular accident | CAD, Smoker |
| 2 | 75 | M | 29 | Cerebrovascular accident | CAD |
| 3 | 64 | M | 36 | Cerebrovascular accident | Hyperlipidema |
| 4 | 55 | M | 42 | Cerebrovascular accident | Smoker |
| 5 | 44 | F | 93 | Cerebrovascular accident | None |
| 6 | 66 | M | 49.5 | Aspiration, anoxic encephalopathy | CAD |
| 7 | 50 | M | 49 | Head trauma secondary to motor vehicle accident | Diabetes |
|  |  |  |  |  |  |
| **Control Diaphragms** | | |  | **Thoracic Surgery** |  |
| 1 | 71 | F |  | Lung Cancer | COPD, CAD, HTN |
| 2 | 71 | F |  | Lung Cancer | COPD, Colon Cancer |
| 3 | 49 | F |  | Lung Cancer | Smoker |
| 4 | 74 | F |  | Lung Cancer | ILD, HTN, Diabetes, Aortic Stenosis |
| 5 | 72 | F |  | Esophageal Cancer | GERD, Colon Cancer |
| 6 | 71 | F |  | Lung Cancer | COPD, Colon Cancer |
| 7 | 62 | M |  | Bronchiectasis | Lymphoma |
| 8 | 57 | F |  | Benign Vascular Tumor | Breast Adenoma |
|  |  |  |  |  |  |
| **Control Lungs** | | |  | **Thoracic Surgery *** |  |
| 1 | 80 | F |  | Lung Cancer | COPD, Atrial Fibrillation |
| 2 | 78 | F |  | Lung Cancer | Never smoker |
| 3 | 27 | M |  | Liposarcoma | Never smoker |
| 4 | 60 | M |  | Lung Cancer | COPD, HTN, Diabetes |
| 5 | 70 | F |  | Lung Cancer | COPD, Hypothyroidism |
| 6 | 84 | F |  | Lung Cancer | HTN, Never smoker |

CAD=Coronary artery disease, COPD=Chronic obstructive pulmonary disease, HTN=Hypertension, ILD=Interstitial lung disease, GERD=Gastroesophageal reflux disease

*Lung tissue for the analysis was obtained from areas not involved by tumor
