# Supplementary material for: Characterization of SARS‐CoV‐2 Entry Genes in Skeletal Muscle and Impacts of In Vitro Versus In Vivo Infection
Source: J Cachexia Sarcopenia Muscle. 2025 Jan 27;16(1):e13705. doi: 10.1002/jcsm.13705 (PMC11772215; doi:10.1002/jcsm.13705)
Supplement: Supplementary file 5 — Table S2 PCR Primer Sequences [file JCSM-16-e13705-s001.docx]

**Supplemental Table 2: PCR Primer Sequences**

| **Human** | | | | |
| --- | --- | --- | --- | --- |
| *Gene name* | | *Forward sequence (5’-3’)* | | *Reverse sequence (5’-3’)* |
| β-Actin | | CTCTTCCAGCCTTCCTTCCT | | AGCACTGTGTTGGCGTACAG |
| ACE2 | | TCCATTGGTCTTCTGTCACCCG | | AGACCATCCACCTCCACTTCTC |
| TMPRSS2 | | ATCGACAAATGAGGACGGCT | | AATCATGCACGGGGAAGCAA |
| Furin | | GCTGGGCTCCATCTTTGTCT | | TGTGAGACTCCGTGCACTTC |
| CTSB | | CCGGGCACAACTTCTACAAC | | GGATGCAGATCCGGTCAGAG |
| CTSL | | CATCCTTGCTGCCTTTTGCC | | ACACTGCTCTCCTCCATCCT |
| MuRF1 | | GCCACCTTCCTCTTGACTGC | | GGCGTCTGCTATGTGCTCTA |
| Atrogin1 | | CCTCTGCATGGCAGGAGTAA | | TGTTGAAAGCTTCCCCCAGG |
| LC3B | | AGCAGCTTCCTGTTCTGGAT | | TGAGCTGTAAGCGCCTTCTAA |
| Gabarapl1 | | TGGCATCCTCTAGCCCTTGT | | TCAGAGCCTTACACTGCCAT |
| **Hamster** | | | | |
| *Gene name* | *Forward sequence (5’-3’)* | | *Reverse sequence (5’-3’)* | |
| β-Actin | CCATTGGCAACGAGCGGTT | | ATAGAGGTCTTTGCGGATGTCG | |
| MuRF1 | CCGGGAATGACCGAGTACAG | | GATGGCGTAGAGGGCATCAA | |
| Atrogin1 | CAGTACCACTTCTCCGAGCG | | TAGGGTGTCCCCATACTGCT | |
| LC3B | CGTCGTGGGAGCAGTATCC | | GGGATCTTGGTGGGGTGTTG | |
| Gabarapl1 | CTATCCCTCCCACGAGTGCT | | GCTTCGGCCTCATTTCCCAT | |
| P62 | CATCAGAGGATCCCAGTGTCAAT | | TCTTCCCTCCATGTTCCACATC | |
| **SARS-CoV-2** | | | | |
| *Gene name* | *Forward sequence (5’-3’)* | | *Reverse sequence (5’-3’)* | |
| UpE | ATTGTTGATGAGCCTGAAG | | TTCGTACTCATCAGCTTG | |
